# 

**Published:** 1897-07

**Authors:** 


Vol. XVIII. JULY, 1897. No. 7.

PUBLISHED MONTHLY AT $3 A YEAR. SINGLE COPIES, 30 CENTS.

LIBRARY
Indexed
S.G.O.

### THE JOURNAL OF COMPARATIVE MEDICINE AND VETERINARY ARCHIVES

EDITED AND PUBLISHED BY
RUSH SHIPPEN HUIDEKOPER, Veterinarian (Alfort),
W. HORACE HOSKINS, D.V.S.,
H. D. GILL, V.S.

TWENTY-FOUR PAGES EXTRA THIS MONTH.

### CONTENTS.

### UNITED STATES VETERINARY MEDICAL ASSOCIATION.

**Proceedings of the Thirty-third Annual Meeting held at Buffalo, N. Y., September, 1896. (Concluded.)**

Some Poisonous Stock Foods. By N. S. MAYO, V.S. . . . 225
Toe-clip Injuries. By JAMES A. WAUGH, V.S. . . . 227
Dairy Inspection. By FRED. BRAGINTON, V.S. . . . 231
Index . . . 237
Contents, Officers, Committees, and Members . . . i–xxiv

ALL COMMUNICATIONS AND PAYMENTS SHOULD BE ADDRESSED TO
**Dr. W. Horace Hoskins, Managing Editor, 3452 Ludlow St., Philadelphia, Pa.**
Copies may be had on order of all news-dealers. In London of Baillière, Tindall & Cox.

